# Uterine and placental blood flow indexes and antinuclear autoantibodies in unexplained recurrent pregnancy loss: should they be investigated in pregnancy as correlated potential factors? A retrospective study

**DOI:** 10.1186/s12884-020-2724-6

**Published:** 2020-01-20

**Authors:** Valentina Bruno, Carlo Ticconi, Federica Martelli, Marzia Nuccetelli, Maria Vittoria Capogna, Roberto Sorge, Emilio Piccione, Adalgisa Pietropolli

**Affiliations:** 1 Academic Department of Biomedicine and Prevention, Section of Gynecology, University of Rome “Tor Vergata”, Via Montpellier, 1 –, 00133 Rome, Italy; 2 Academic Department of Surgical Sciences, Section of Gynecology, Tor Vergata University Hospital, Viale Oxford, 81 –, 00133 Rome, Italy; 3 Department of Experimental Medicine and Surgery, Tor Vergata University Hospital, Viale Oxford, 81 -, 00133 Rome, Italy; 4 Department of Systems Medicine, Laboratory of Biometry, University of Rome “Tor Vergata”, Via Montpellier, 1 –, 00133 Rome, Italy; 5 Academic Department of Systems Medicine, Section of Gynecology, University of Rome “Tor Vergata”, Via Montpellier, 1 –, 00133 Rome, Italy

**Keywords:** uRPL, ANA, VOCAL, Placental blood flow supply, LMWH

## Abstract

**Background:**

The potential role of antinuclear antibodies (ANA) in recurrent pregnancy loss (RPL) pathogenesis is still debated, although some evidences suggest that they could affect pregnancy outcome, leading to a higher miscarriage rate in these patients. A hypothesized mechanism is through changes in uterine flow in pre-conceptional stage, by modifying endometrial receptivity in RPL. However, scant data are available, in pregnancy, about their role in RPL placental perfusion, also in relation to its potential treatments, such as low molecular weight heparin (LMWH). The aim of this study is to retrospectively further investigate the correlation between two-dimensional (2D) and three-dimensional (3D) uterine and placental flow indexes and the presence or the absence of ANA in women with unexplained RPL (uRPL), treated or not treated with LMWH.

**Methods:**

2D Doppler measurement of pulsatility index (PI) of the uterine arteries and 3D ultrasonography determination of vascularization index (VI), flow index (FI) and vascularization flow index (VFI) was carried out with the aid of the virtual organ computer-aided analysis (VOCAL) technique in LMWH treated (n 24) and not treated-uRPL patients (n 20) and in the relative control group (n 27), each group divided in ANA+ and ANA- subgroups. Serum assay for the presence of ANA was performed in all women.

**Results:**

No differences were found in PI, VFI and VI values, by comparing the different groups. A difference in VI values was found for ANA- patients between RPL women not treated with LMWH and the treated ones (*p* = 0,01), which have lower VI values and similar to controls. By considering only ANA- treated and not treated RPL patients, the ROC curve shows an area of 0,80 and at the VI cut-off of 11,08 a sensitivity of 85% and a specificity of 67%.

**Conclusions:**

LMWH could exert a potential beneficial effect in restoring the physiological blood flow supply in terms of VI in uRPL ANA- status, suggesting to include ANA and VI investigations in the RPL diagnostic algorithm in a research context, since further studies are needed to clarify this challenging hypothesis in order to try to ameliorate ANA and abnormal placental vascularization negative influence on RPL pregnancy outcome .

## Background

Recurrent pregnancy loss (RPL) is defined as the loss of two or more pregnancies before the 24th week of gestation [1]. Known causes of RPL include: parental chromosomal abnormalities, uterine abnormalities, metabolic and endocrinological factors, immunological factors, major thrombophilia, autoimmune diseases and antiphospholipid syndrome [1, 2]. These abnormalities, ranging from genetic, anatomical, metabolic and endocrinological factors to autoimmune diseases explain only 50–60% causes of RPL, conversely 40–50% remains unexplained [1]. Among the known causes of RPL related to a systemic autoimmunity, there are some evidences, not clearly elucidated, of the possible role of antinuclear antibodies (ANA) in RPL etiopathogenesis. The value of ANA in identifying patients affected by RPL with potential immune abnormalities, although not fully defined, has already been investigated in previous case-control studies: ANA were found more frequently in RPL patients than in controls [3–7]. ANA could also affect pregnancy outcome in RPL women, leading to a higher miscarriage rate in ANA positive (+) women, compared to ANA negative (−) ones [8]. Furthermore, ANA+ RPL women, who exhibit negative ANA in early pregnancy, were likely to have an ongoing pregnancy, in contrast to those patients who were still positive in the first trimester [9]. However, in spite of these studies, the role of ANA in women with RPL is still a debated subject. There are some experimental data that suggests the existence of a possible correlation between ANA and changes in the uterine blood flow in non pregnant women affected by RPL [10–12]: the uterine perfusion, in fact, regulates the endometrial receptivity and its alteration might be associated with pregnancy complication at an early stage. Conversely, scant data are available, in pregnancy, about the role of ANA in placental perfusion in relation to RPL and its potential medical treatments, such as low molecular weight heparin (LMWH), which is empirically used in clinical practice (even if not recommend in the European Society of Human Reproduction and Embryology -ESHRE guidelines) [1] for other potential therapeutic properties: prevention of trophoblast apoptosis, enhancement of trophoblast invasiveness, improvement of the endothelial and vascular environments, regulation of embryo implantation, immune functions, beyond its well known anticoagulant effects and inhibitory action on the complement system [13].

## Methods

### Objectives of the study and study design

The aim of the present pilot retrospective study was to investigate the effects of ANA on uterine artery flow and on placental flow and vascularization in RPL pregnant patients, also in relation to LMWH assumption, since this treatment could potentially exert beneficial effects in this subgroup of women. In fact, to our knowledge no data are available concerning LMWH effect on uterine and placental flow and vascularization indexes. This purpose was achieved by combining two-dimensional (2D) ultrasound technique and three-dimensional (3D) power Doppler imaging with the virtual organ computer-aided analysis (VOCAL) technique, in order to obtain a higher sensitivity. Indeed, it has been recently suggested that 3D ultrasound application to assess placental vascularization could be used an accurate and reproducible technique to further investigate the adequacy of the placentation process [14–17].

### Subjects of the study

The present study involved 27 control pregnant women (11 ANA- and 16 ANA+), 20 RPL pregnant women not taking any kind of treatment (10 ANA- and 10 ANA+) and 24 RPL pregnant women treated with LMWH (10 ANA- and 14 ANA+) (age range: 27–43 years).

All RPL women, referred to the RPL Unit of Tor Vergata University Hospital – Rome, underwent the standardized diagnostic workup, performed before pregnancy:

1. Clinical diagnosis: a careful anamnesis has been carried out at different levels (familiar, physiological, pathological). Miscarriages occurred in previous pregnancies were checked by analyzing all clinical documents of previous pregnancies, such as quantitative serum human chorionic gonadotropin (hCG) assays, ultrasound documentation and clinical records.
2. Gynecological physical examination;
3. Transvaginal ultrasound and a 3D pelvic ultrasound;
4. Hysteroscopy and endometrial biopsy;
5. Endocrinological evaluation: assay of luteinizing hormone (LH), follicle-stimulating hormone (FSH), prolactin, progesterone in the luteal phase, thyroid stimulating hormone (TSH), free tri-iodothyronine (FT3), free thyroxine (FT4), pituitary and ovarian androgens, insulin and glucose curve;
6. Rating of maternal and paternal set of chromosomes;
7. Rating of immune condition: anti-phospholipid antibodies, lupus anticoagulant, anti-β2GPI and anti-annexin V, anti-thyroid antibodies, extractable nuclear antigen (ENA), anti-double strand DNA (anti-ds DNA), anti-smooth muscle antibody (ASMA) and anti-mitochondrial antibodies (AMA);
8. Thrombophilia screening: protein C, protein S, antithrombin III (AT III), activated protein C resistance (APCR), homocysteine;
9. Determination of the following mutations: factor V [G1691A Leiden], factor II prothrombin [G20210 A], plasminogen activator inhibitor [PAI-1 4G/5G], methylen tetrahydrofolate reductase [MTHFR C677T and A1298C].

The diagnostic work-up aims to identify proven, probable and dubious causes of RPL [18].

All women in which one of the previous factor was detected, were excluded; only unexplained RPL women (uRPL) were included in the study.

This study was carried out in a period of time ranging from 2014 to 2018: LMWH empirical use (Clexane® 4000 I.U. from the first beta-HCG positive assay for all the pregnancy course) in uRPL pregnant women was limited in the last 3 years, since evidence-based level has not yet been reached to prove its beneficial use in case of unexplained RPL [1]. Thus, all patients enrolled in this study treated with LMWH were the earliest collected ones and their clinical characteristics matched with the non-treated patients enrolled later on in the collecting process.

Control pregnant women of childbearing age, which match the major clinical characteristics (age and body mass index) with those of RPL women, were enrolled. They had to fulfil the following criteria: a medical history of least 2 or more normal pregnancies at term without having any miscarriage. Patients with chronic disease and/or who developed a complication, such as preeclampsia, gestational hypertension, preterm labour, gestational diabetes, later in pregnancy were excluded from the study. These patients were followed at the University Hospital of Tor Vergata Clinic for physiological pregnancies, according to the standardized protocol used in our unit, which complies with the NICE Clinical Guidelines [19]. Peripheral blood samplings were performed in all patients to check their ANA status (positivity or negativity), only after a written informed consent.

All women included in the study underwent the study protocol, which consisted in: 1) measurements of uterine artery pulsatility index (PI) by 2D ultrasound; 2) 3D power doppler ultrasound investigation to evaluate the vascularization index (VI), flow index (FI), vascularization flow index (VFI). All pregnancies involved in the study were at term, without any pregnancy complications, in order to avoid bias in placental flow indexes assays [15, 17].

### Transabdominal Doppler ultrasound

Placental flow indexes (VI, FI, VFI) and the velocimetry of the uterine arteries (PI) were assessed, between the 11 + 0 and the 13 + 6 weeks of gestation, since this period correspond to the gestational weeks in which both the routinely performed first trimester ultrasound scan [20] and since the majority of miscarriages in RPL women occur within 14 weeks [21].

#### Acquisition of 2D Doppler velocity waveforms

The PI of the left and right arteries were measured by a Voluson E6 GE ultrasound machine (GE Healthcare, Kretztechnik, Zipf, Austria), equipped with a 3,7 MHz 3D abdominal transducer with colour imaging capabilities (Fig. 2). A 7,5 MHz 3D transducer has been used for the transvaginal evaluation. All measurements were entered prospectively into a computer database for offline analysis.

#### Acquisition of 3D placental vascular indexes

**Fig. 1:**
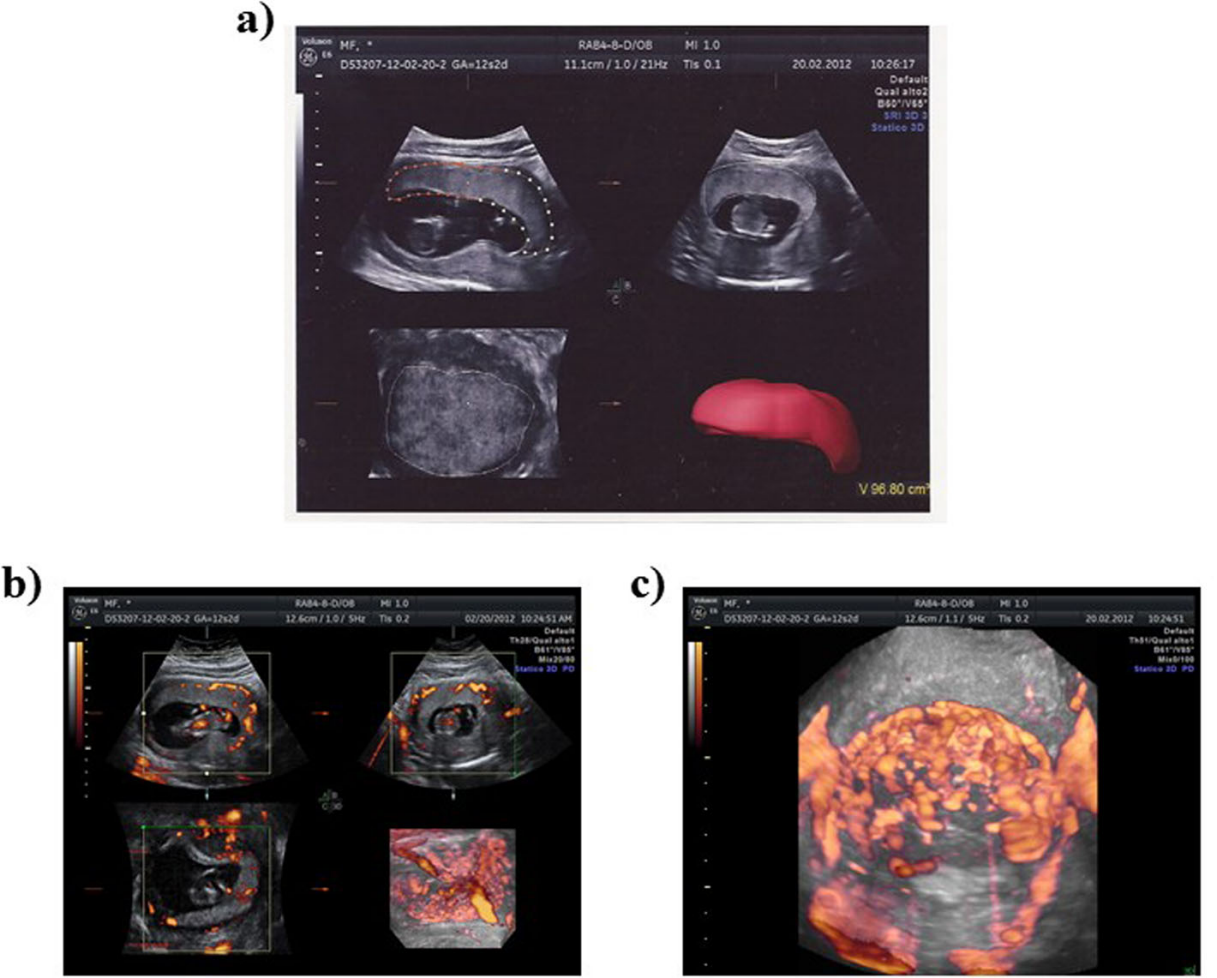
3D power Doppler acquisition images. **a** 3D power Doppler image of a placenta at 12 + 2 weeks in a multiplanar mode. Placenta rotated horizontally. Image shows the placental bed after tracing. **b** Sampling of placental volumes by 3D sonography: Two dimensional ultrasound and Power Doppler imaging of the placental vessels. **c**: 3D volume rendering

Ultrasound 3D volume scanning were performed by 2 of the authors (FM and MVC) with a 4-to 8-MHz transabdominal transducer and a 5–10-MHz transvaginal probe using a Voluson E6 GE ultrasound machine (GE Healthcare, Kretztechnik, Zipf, Austria). In all cases the same instrument settings was used (angio mode, cent; smoothing, 4/5; FRQ, low; quality, 16; density, 6; enhancement, 16; balance, G > 150; filter, 2; actual power, 2 dB; pulse repetition frequency, 0.9 kHz). Power Doppler ultrasound was applied to the image the placental vascularization. The 3D placental volume was acquired, keeping the probe in a longitudinal position perpendicular to the abdomen. The sweep angle was set at 85°. All volumes were stored and later analyzed with the Virtual Organ Computer-Aided Analysis II program (GE Healthcare, Milwaukee, WI). The contour mode in the Virtual Organ Computer-Aided Analysis II program was set to manual. The rotation steps were set at 15°, 18 and 12 contours of the placenta were drawn manually after a 15° rotation from the previous one as automatically performed by the software. Once all contours had been drawn, the volume of the placenta was automatically calculated. Then, placental vascularization were recorded. Later on, each volume was analyzed to calculate the placental vascularization indexes (VI, FI, VFI) (Fig. 1). Intra- and interobserver agreement evaluation was performed trough an independent and separate analysis of the 3D Power Doppler placental vascular indices by the same operator (FM) and by a second obstetrician (MVC); a 0.80 correlation or higher has been shown in all analysis, in line with the reproducibility data on this technique shown in the most recent scientific literature [ 14, 22].

### ANA assay

Peripheral blood samples were collected in BD Vacutainer tubes, centrifuged and sera stored at − 20 °C until the assay. ANA detection was performed with an indirect immunofluorescence method (IIF) based on the use of Hep − 2 cells, a human epithelial cell line, as a substrate (INOVA Diagnostics, San Diego, CA, USA). This substrate provides cells in different stages of development, and mitotic figures aid in differential pattern recognition. Specimens were diluted with phosphate-buffered saline with a 1:80 screening dilution. Slides were examined by two observers with a fluorescence microscope at × 40 magnification. Negative and positive controls with known antibody titers were used for quality control.

### Statistical analysis

All data were initially entered into an EXCEL database (Microsoft, Redmond, Washington – United States) and the analysis was performed by using the Statistical Package for the Social Sciences Windows, version 15.0 (SPSS, Chicago, Illinois, USA). Descriptive statistics consisted of the mean ± standard deviation (mean ± sd), since all considered parameters have a normal distribution (after confirmation with histograms and the Kolgomorov-Smirnov test). Statistical comparison has been carried Anova one-way test or Anova two factor with the post-hoc test of Bonferroni, since data were normally distributed. For significant results found in the statistical analysis comparison described above, a ROC curve was ran to evaluate specificity and sensitivity of the vascularization parameters included in the study. A *p* value of < 0.05 was considered statistically significant. All graphs were produced with Excel or SPSS.

## Results

### Clinical characteristics

No significant differences were detected in patients’ age and body mass index, irrespective of ANA status, of the presence of RPL, and of the treatment with LMWH (Table 1). Furthermore, no significant differences were found in number of miscarriages as well as in the gestational age at which previous miscarriages occurred between uRPL ANA+ and uRPL ANA- women, irrespective of the LMWH therapy (*p* > 0.05) (Table 1). All women enrolled in this study had at term pregnancy. No differences were found in term of birth weight and gestational week of the delivery, and blood pressure among all the different study groups.

**Table 1:** Clinical characteristics of studied patients

	RPL (*n* = 20)		RPL (*n* = 24)		Controls (*n* = 27)		One-way ANOVA	
	Not-treated		LMWH-treated					
	ANA - (n = 10)	ANA + (n = 10)	ANA - (*n* = 10)	ANA + (n = 14)	ANA - (n = 11)	ANA + (n = 16)	F	*P* value
Age (years)	34 + 5	35 + 6	36 + 4	35 + 5	36 + 2	36 + 3	0.4	ns
BMI (Kg/m^2^)	25 + 4	26 + 5	24 + 4	24+ 5	24 + 3	26 + 2	0.78	NS
Number of miscarriages	3 + 0.9	3 + 1	2.9 + 0.8	3.1 + 0.8	–	–	0.1	NS
Week of miscarriage	8.4 + 2	8.7 + 2.6	8.5 + 2	9 + 2.5	–	–	0.16	NS
Blood pressure	97,2 / 73,2	108,7 / 75,7	109,7 / 77,2	110,2 / 78,07	113,1 / 74,91	104,3 / 77,75	1,08/1,7	NS
gestational week of the delivery	39,1 + 1,1	39,2 + 1,8	39,4 + 0,97	39,2 + 1,48	39,9 + 0,94	39,4 + 1,26	0,87	NS
Birth weight	**3228** + **269,2**	**3308** + **287**	**3436** + **313,5**	**3233** + **358,3**	3279 + 368,0	3241 + 287,6	0,34	NS

Data are expressed as Mean + SD or mean only

*ANA* antinuclear antibodies; *RPL* recurrent pregnancy loss; *BMI* body mass index; *NS* not significant; *ONE-WAY ANOVA* one-way analysis of variance

### Uterine arteries flow, vascularization indexes and antinuclear antibodies status

2-D and 3-D Power Doppler indexes values obtained for each group and subgroup are reported in Table 2.

**Table 2:** 2-D and 3-D Power Doppler Indexes values obtained for each group and subgroup

Control women			Not-treated RPL women		LMWH-treated RPL women	
	ANA-	ANA+	ANA-	ANA+	ANA-	ANA+
PI	1.35 ± 0.52	1.16 ± 0.43	1.12 ± 0.21	1.31 ± 0.46	1.37 ± 0.48	1.26 ± 0.42
FI	42.46 ± 2.81	40.53 ± 4.39	43.24 ± 8.46	38.71 ± 6.97	44.18 ± 6.85	46.22 ± 4.57
VFI	5.41 ± 2.05	6.34 ± 4.51	9.31 ± 2.57	5.13 ± 2.1	4.93 ± 2.94	6.91 ± 5.32
VI	12.79 ± 4.76	15.31 ± 9.3	20.35 ± 6.16	13.35 ± 5.32	8.61 ± 5.39	11.11 ± 4.09

Values of PI, FI, VI and VFI ​​obtained for each group and subgroup. Data are expressed as Mean + S.D.

No significant differences could be detected in the PI values of the left and right uterine arteries in all women. Therefore, the impedance to uterine artery blood flow was reported in terms of the average PI values.

**Fig. 2:**
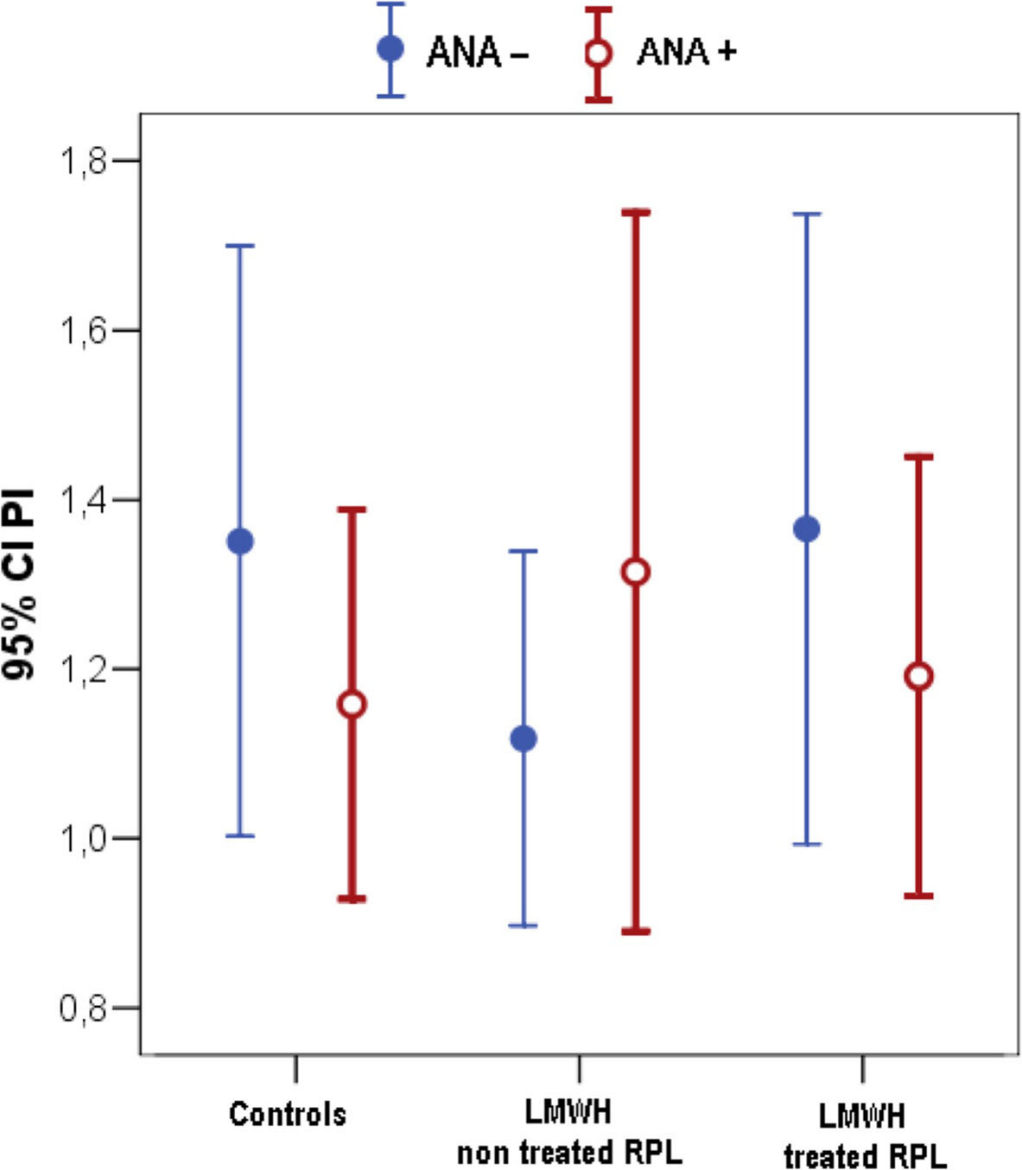
2D ultrasound analysis of uterine flux index (PI). PI values detected in ANA- (*n* = 11) and ANA+ (*n* = 16) control pregnant women, ANA- (*n* = 6) and ANA+ (*n* = 7) RPL pregnant patients not treated with LMWH, ANA- (*n* = 9) and ANA+ (*n* = 14) RPL pregnant patients treated with LMWH. Data are expressed as means ± SD. ANOVA two factors followed by Bonferroni’s post-hoc test: n.s. PI = pulsatility index

Two-D ultrasound analysis of uterine flow indexes showed that the PI did not differ between all different groups (Fig. 2).

**Fig. 3:**
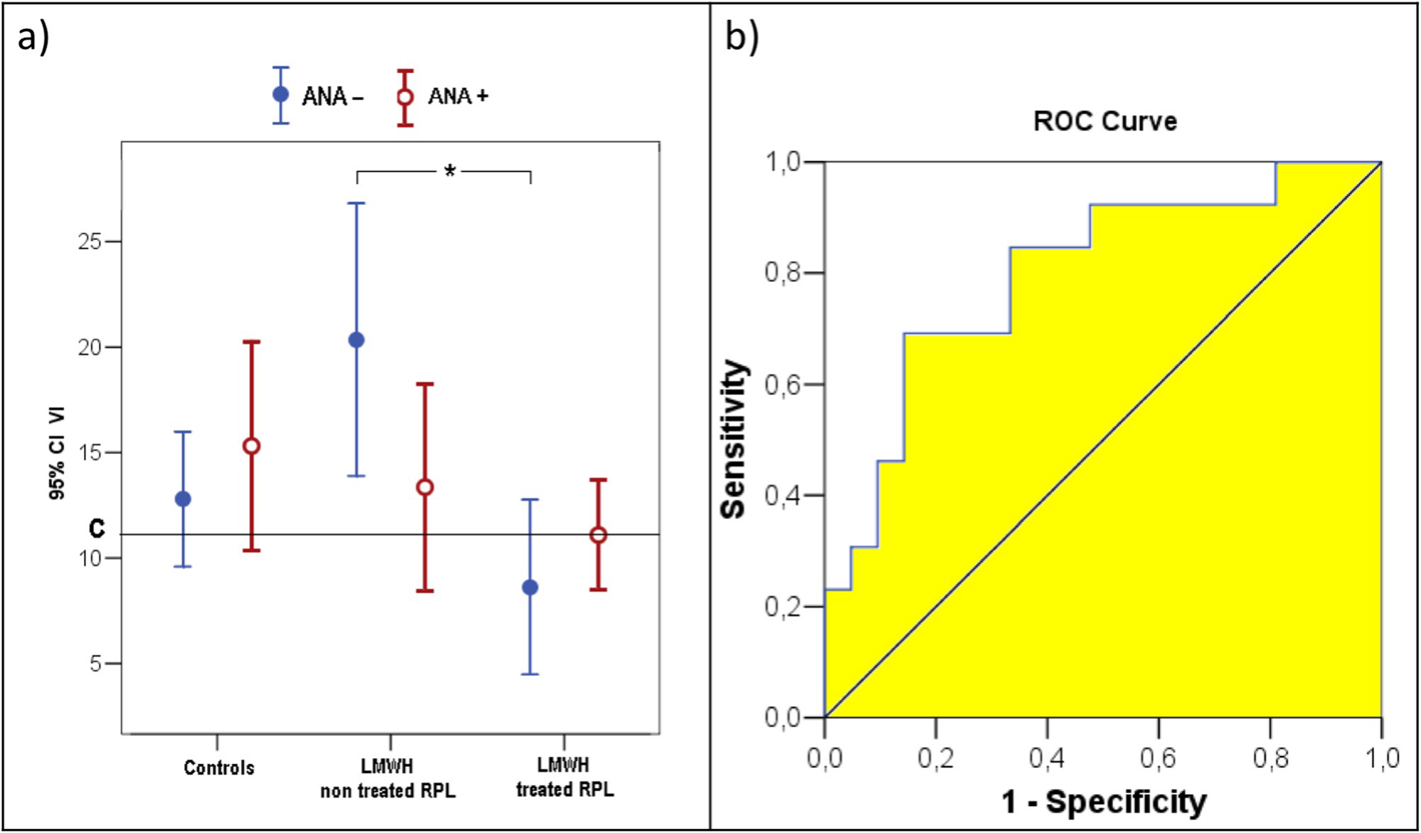
3D ultrasound analysis of VI index. **a**. VI values detected in ANA- (*n* = 11) and ANA+ (*n* = 16) control pregnant women, ANA- (*n* = 6) and ANA+ (*n* = 7) RPL pregnant patients not treated with LMWH, ANA- (*n* = 9) and ANA+ (*n* = 14) RPL pregnant patients treated with LMWH. Data are expressed as means ± SD. ANOVA two factors followed by Bonferroni’s post-hoc test. (*) Bonferroni’ s test *p* = 0,01. VI = vascularisation index. C = VI cut-off determined at the ROC curve: 11,08. **b**. ROC curve: area 0,80; VI cut-off determined 11,08; sensitivity 85% and specificity 67%

Three-D ultrasound analysis of uterine flow and vascularization indexes revealed that there is a statistical significant difference in VI values for ANA- patients between RPL women not treated with LMWH (16,6 ± 6,6) and the treated ones (10 ± 4,7), which have lower VI values and similar to controls (14,3 ± 7,8). Conversely, there are not significant differences between all ANA+ groups (Fig.3a).

By considering only ANA- treated and not treated patients, the ROC curve shows an area of 0,80 and at the VI cut-off of 11,08 a sensitivity of 85% and a specificity of 67% (Fig. 3b).

**Fig. 4:**
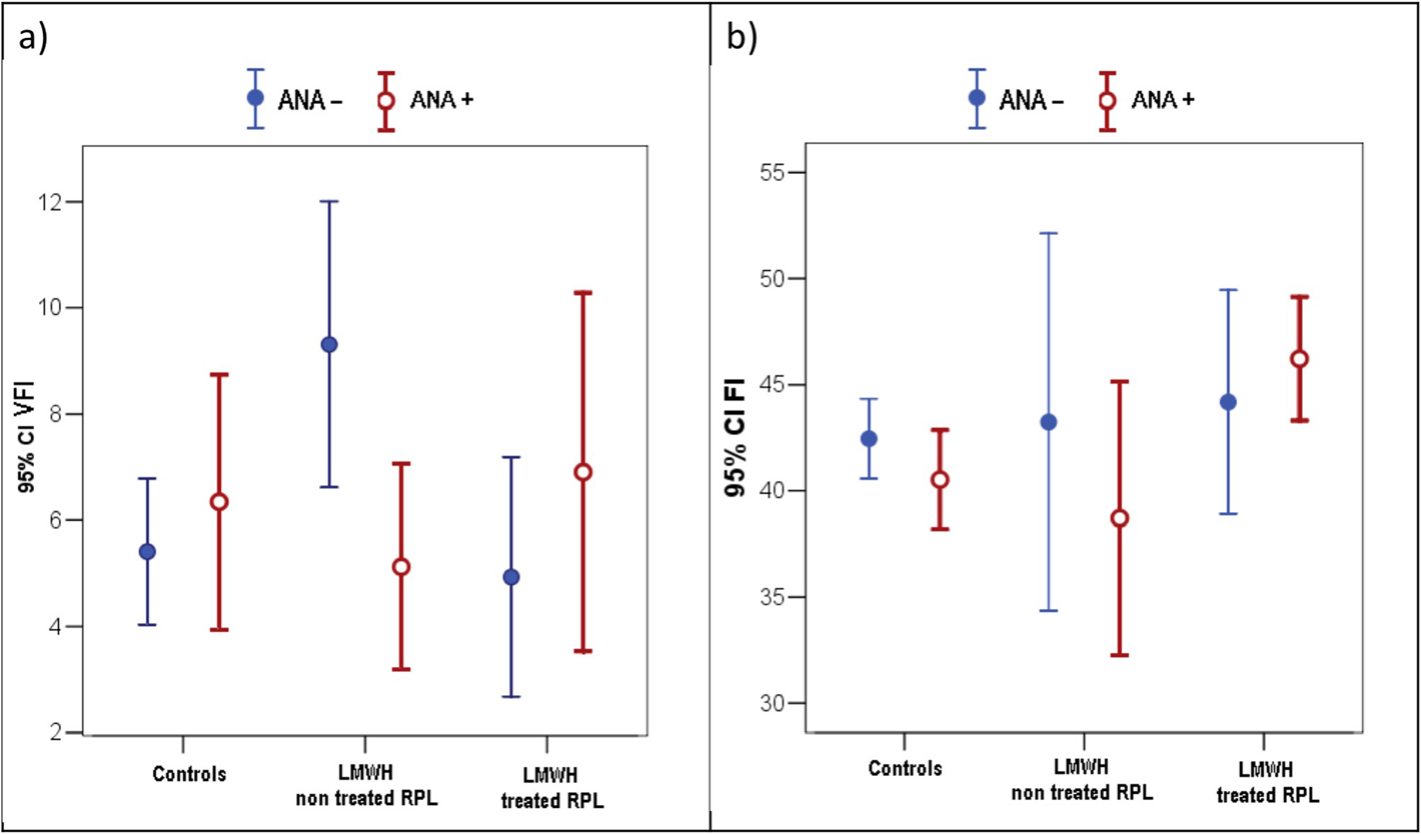
3D ultrasound analysis of VFI and FI indexes. **a**. VFI and **b**) FI values detected in ANA- (*n* = 11) and ANA+ (*n* = 16) control pregnant women, ANA- (*n* = 6) and ANA+ (*n* = 7) RPL pregnant patients not treated with LMWH, ANA- (*n* = 9) and ANA+ (*n* = 14) RPL pregnant patients treated with LMWH. Data are expressed as means ± SD. ANOVA two factors followed by Bonferroni’s post-hoc test: n.s. VFI = vascularisation flow index; FI = flow index

There are no statistically significant differences in VFI between all groups, even if the LWMH-non treated ANA- RPL group show a higher mean compared to all other group (Fig. 4a).

There are no statistically significant differences in FI between all groups (Fig. 4b).

## Discussion

Despite the PI of the uterine artery has previously been showed to have significantly increased values in women with RPL [10, 11], and it is even higher in RPL ANA+ patients compared to ANA- patients [10], in a subsequent study carried out by the same authors it is clear that in pregnant women affected by RPL the PI of the uterine arteries, detected at 4–5 weeks of gestation, could be an ANA independent-RPL index [23]. Our findings regarding the PI values in pregnant RPL women, irrespective to ANA status are in accordance with the previous literature data.

In our study VI resulted significantly higher in RPL patients not treated with LMWH, but only in an ANA- status. Therefore, if we consider the effect of LMWH in these patients, it seems to adjust VI value in RPL, leading this parameter to values closer to the control group. Taken together, the above results suggest that the presence of ANA could induce other modifications in placental vascularization, for which a potential treatment with LMWH could be ineffective. Since VI is an index of the density of blood vessels in the analyzed volume, its alteration in women with RPL could reflect a possible alteration in placental vascularity in these subjects. LMWH can ameliorate placental vascularization in RPL women, leading this subgroup of patient in the same clinical condition of control women. The mechanisms of LMWH treatment should be further elucidated in subsequent focused studies.

Furthermore, in our analysis by applying the ROC curve analysis, 11,08 appears to be the VI value which could considered as a good cut-off, to guarantee a sensitivity of 85% and a specificity of 67% in discriminating when an indication for LMWH treatment could be proposed in order to lead VI value closer to control women. However, a sensitivity of 85% and a specificity of 67% cannot be accepted as a diagnostic tool, but our results remains an informative contribution within the RPL item, to potentially plan further randomized clinical trials (RCTs) to investigate the possible clinical application of this parameter.

The omogenous VI values in case of ANA+ in all the three groups shows that the behavior in the VI parameter does not change, independently of RPL status. It could be hypothesized that ANA+ status does not induce a more severe condition in RPL when compared to controls in terms of VI and, therefore in placental vascularization.

At the best of our knowledge this is the first study showing an effect of LMWH on placental VI index in relation to RPL and/or ANA status, even though the study population is low and further investigations should be considered in a larger cohort of patients. Previous data available in this field about RPL treatment was about low-dose aspirin (100 mg / day) alone or in association with Omega-3, which has been investigated in relation to uterine arteries flow indexes in non-pregnant women. It has been shown that this kind of treatments are able to improve the uterine perfusion with a reduction of the PI of the uterine arteries in the mid- luteal phase in patients with RPL [24].

LMWH effects, described from our results, could have important clinical applications in terms of ameliorating placental vascularization, but its use should be targeted according to VI value and ANA status to be effective.

RCTs with higher numbers of enrolled women are needed to further elucidate our research hypothesis, since the observed findings deserve further elucidation, due to the importance of ANA+ status on RPL negative pregnancy outcome shown by the most recent scientific literature, included in RPL ESHRE guidelines 2017 [25].

Furthermore, several study in the literature, have pointed out the power Doppler imaging limitations in detecting and reproducing organ vascularity, due to not proper US machine settings, such as pulse repetition frequency (PRF) and wall motion filter (WMF) [26]. This problem, although with minor impact on placental vascularization, should be taken into account for data interpretation and discussion.

## Conclusion

In conclusion, LMWH could exert a potential beneficial effect in restoring the physiological blood flow supply in terms of VI in uRPL ANA- status, which could be related to a condition of impairment in placental blood flow supply, suggesting to include ANA and VI investigations in the RPL diagnostic algorithm in a research context.

## Data Availability

The datasets used and analysed during the current study are available from the corresponding author on reasonable request.

## Abbreviations

(IIF) method: Indirect immunofluorescence
2D: Two-dimensional
3D: Three-dimensional
AMA: Anti-mitochondrial antibodies
ANA: Antinuclear antibodies
anti-ds DNA: Anti-double strand DNA
APCR: Activated protein C resistance
ASMA: Anti-smooth muscle antibody
AT III: Antithrombin III
ENA: Extractable nuclear antigen
ESHRE: European Society of Human Reproduction and Embryology
FI: Flow index
FSH: Follicle-stimulating hormone
FT3: Free tri-iodothyronine
FT4: Free thyroxine
hCG: Human chorionic gonadotropin
LH: Luteinizing hormone
LMWH: Low molecular weight heparin
mean ± sd: Mean ± standard deviation
MTHFR C677T and A1298C: Methylen tetrahydrofolate reductase
PAI-1 4G/5G: Plasminogen activator inhibitor
PI: Pulsatility index
PRF: Pulse repetition frequency
RCTs: Randomized clinical trials
RPL: Recurrent pregnancy loss
TSH: Thyroid stimulating hormone
uRPL: Unexplained recurrent pregnancy loss
VFI: Vascularization flow index
VI: Vascularization index
VOCAL: Virtual organ computer-aided analysis
WMF: Wall motion filter
